# Cysteine proteinase inhibitor cystatin C in squamous cell carcinoma of the head and neck: relation to prognosis

**DOI:** 10.1038/sj.bjc.6601830

**Published:** 2004-04-27

**Authors:** P Strojan, I Oblak, B Svetic, L Šmid, J Kos

**Affiliations:** 1 Department of Radiotherapy, Institute of Oncology, SI-1000 Ljubljana, Slovenia; 2 Department of Biochemistry, Institute of Oncology, SI-1000 Ljubljana, Slovenia; 3 University Department of Otorhinolaryngology and Cervicofacial Surgery, Clinical Center, SI-1000 Ljubljana, Slovenia; 4 Department of Pharmaceutical Biology, Faculty of Pharmacy, University of Ljubljana, SI-1000 Ljubljana, Slovenia; 5 Department of Biochemical Research and Drug Design, KRKA, d.d., SI-1000 Ljubljana, Slovenia

**Keywords:** cystatin C, head and neck cancer, squamous cell carcinoma, prognosis

## Abstract

To determine the role of the cysteine proteinase inhibitor cystatin C in the invasive behavior of squamous cell carcinoma of the head and neck (SCCHN), Cystatin C protein level was measured in 82 pairs of primary tumour tissue and adjacent noncancerous mucosa, using the enzyme-linked immunosorbent assay. The median level of cystatin C in tumour tissue was 1.18 times lower than that in corresponding mucosa (*P*=0.031). In normal mucosa samples, the cystatin C level was influenced by the site of sampling: it was lower in nonlaryngeal tissue samples (oral cavity, oro- or hypopharynx) than in laryngeal samples (*P*=0.004). The tumour cystatin C level correlated inversely with pN-stage (*P*=0.047), whereas a trend of lower cystatin C levels was observed in the group with extranodal tumour extension compared to those with no extranodal spread (*P*=0.069). In univariate analysis, the patients with low tumour cystatin C levels exhibited poor disease-free survival (DFS, *P*=0.013) and disease-specific survival (DSS, *P*=0.013). In multivariate analysis, the most powerful predictor of survival was pN-stage (DFS: *P*=0.040, HR 2.78; DSS: *P*=0.011, HR 4.36,), followed by the cystatin C level (DFS: *P*=0.043, HR 0.22; DSS: *P*=0.067, HR 0.25). When comparing the prognostic strength of cystatin C to that of stefin A, another cysteine proteinase inhibitor, which emerged as the most significant prognosticator for survival in our previous study analysing the same cohort of patients, stefin A proved to be significantly more reliable predictor for both DFS and DSS than cystatin C. Our results indicate that cystatin C is implicated in the invasive behavior of SCCHN, and that there are variations in regulation of proteolytic pathways under nonmalignant conditions, inherent to individual subsites inside the upper aerodigestive tract. The correlation between high cystatin C levels and improved survival concurs with the concept of the protective role of high levels of cysteine proteinase inhibitors in tissue homogenates that has been previously suggested by the survival results in breast and lung carcinoma as well as SCCHN.

Cystatin C is a nonglycosylated 13 kDa basic protein, consisting of 120 amino acids. It belongs to the cystatin superfamily of cysteine proteinase inhibitors. Cystatin C is produced at a constant rate by all nucleated cells as a preprotein with a hydrophobic leader sequence of 26 amino acids, indicating an extracellular function of the inhibitor. Cystatin C protein is a product of the *CST*3 gene, which is located on the short arm of chromosome 20. It is freely filtered in the glomeruli and almost completely reabsorbed and catabolised in the proximal renal tubular cells (Newman, 2002). The main physiological role of cystatin C is believed to be the regulation of cysteine proteinases secreted from the cells or leaked from the lysosomes during necrotic or apoptotic processes, which links cystatin C with the aetiology of various diseases, including cancer (Sloane *et al*, 1994).

Immunohistochemically, cystatin C protein was stained intracellularly in the cytoplasmic compartment of tumour cells in a variety of cancer types (Lignelid and Jacobsson, 1992; Lignelid *et al*, 1997; Yoshimura *et al*, 2000; Gaumann *et al*, 2001; Hansen *et al*, 2001; Yano *et al*, 2001). The mechanisms controlling the intracellular inhibition of cysteine proteinases in endosomes and lysosomes with cystatin C entering the endosomal–lysosomal pathway by endocytotic uptake may include dimerisation (Ekiel and Abrahamson, 1996; Merz *et al*, 1997), proteolytic fragmentation by cathepsin D (Lenarčič *et al*, 1991) or neutrophilic granulocyte elastase (Abrahamson *et al*, 1991). Some of these mechanisms may operate already in the pericellular microenvironment and may restrict the inhibitory function of the secreted cystatin C against the proteolytic activity of cell surface associated and/or secreted enzymes (Křepela *et al*, 1998).

Immunohistochemical quantification of cystatin C expression suggested that the concentration of immunoreactive protein in the cells of some tumour types seems to be below the detection limit. In the study by Lignelid *et al* (1997), for example, only a minority of cystatin C mRNA positive brain and pituitary tumours revealed cystatin C-immunoreactive tumour cells. In a group of 88 meningiomas, Strojnik *et al* (2001) detected no immunostaining for cystatin C in any of the tumours, although Lignelid *et al* (1997) reported a 100% positivity for cystatin C transcripts in meningiomas. Alternatively, in homogenised preparations used for cystatin C mRNA determination, cystatin C expressing noncancerous stromal cells within tumours with otherwise low expression level of cystatin C in their cancer cells may contribute substantially to the total cystatin C mRNA level (Lignelid *et al*, 1997). The same reproach regarding the origin of cystatin C holds true when immunobiochemical methods are used for the determination of cystatin C levels in tissue homogenates. However, a large body of evidence proved the tissue homogenate levels of proteinases and inhibitors of various classes to be of significant clinical relevance (Foekens *et al*, 1998; Look *et al*, 2002).

The role of cystatin C in the invasive behavior of squamous cell carcinoma of the head and neck (SCCHN) has not been investigated so far. The aim of the present study was to determine the levels of cystatin C in the tissue homogenates of SCCHN and its surrounding noncancerous mucosa. As SCCHN is an extremely heterogenous disease, it is little surprise that no factor within the wide spectrum of biochemical and histological factors has yet been identified as reliably predicting the natural course of the disease or its response to therapy (Lal, 1994; Quon *et al*, 2001). Thus, the results of the study have been related to established clinical and histopathological features, considering especially the correlation of individual tumour levels of cystatin C with patients’ survival.

## MATERIALS AND METHODS

### Patients

In total, 82 previously untreated patients with primary operable SSCHN entered the study. Of these patients, five were females and 77 males, ranging in age from 37 to 72 years (median age: 59 years). This cohort of patients was the same as in our previous report, examining the prognostic significance of cysteine proteinases cathepsins B and L and their intracellular inhibitors A and B (Strojan *et al*, 2000).

In all patients, therapeutic surgery was performed. It comprised the resection of the primary tumour, related to the lesion extension, together with a surrounding margin of normal tissue, and removal of the related regional lymph nodes from the neck. Postoperative radiotherapy was applied in 74 patients, because of an advanced stage of disease, extranodal tumour spread, residual growth after surgery, or the presence of neoplastic emboli in the lymphatic vessels. The patients were irradiated on a Cobalt-60 unit or a 5-MV linear accelerator, with a daily dose of 1.8–2.0 Gy, 5 days per week. Radiotherapy doses were adapted to the disease extent and ranged from 50 to 66 Gy (median dose: 56 Gy), It was delivered through two opposing lateral fields to the primary tumour and regional lymph nodes, with spinal cord shielded after a dose of 40–44 Gy. One anterior field was added to cover the lower neck lymphatics.

Tumours were staged according to UICC pTNM classification after histopathological examination of surgical specimens (Sobin and Wittekind, 1997). The histopathological grade was defined according to WHO criteria (Azzopardi *et al*, 1981). Clinical and histopathological characteristics of tumours are shown in Table 1

**Table 1:** Tumour parameters of 82 patients with operable squamous cell carcinoma of the head and neck

*Primary tumour site*					
Oral cavity	14				
Oropharynx	18				
Hypopharynx	10				
Larynx	40				
					
*UICC pTNM-classification*					
	N_0_	N_1_	N_2_	N_3_	Total
T_1_	3	1	2	0	6
T_2_	11	3	13	1	28
T_3_	14	1	9	0	24
T_4_	9	2	13	0	24
Total	37	7	37	1	82
					
*Overall stage*					
Stage_I_	3				
Stage_II_	12				
Stage_III_	19				
Stage_IV_	48				
					
*Histopathological grading*					
Well differentiated, G_1_	3				
Moderately differentiated, G_2_	68				
Poorly differentiated, G_3_	11				
					
*Extranodal extention*^a^					
Negative	15				
Positive	30				
					
*Tumour emboli in lymph node vessels*^a^					
Negative	37				
Positive	8				

a pN_+_ patients only, *n*=45.

.

### Sample collection

From operative specimen, two tissue samples of 200–500 mg, representing matched pairs, were obtained from the tumour and adjacent normal mucosa. Immediately after removal, they were immersed in liquid nitrogen until homogenate preparation. Fat and necrotic parts were carefully removed. Pulverisation was performed on the frozen tissue with a Mikro-Dismembranator (Braun, Melsungen, Germany) for 60 s at maximum power, and the resulting tissue powder was suspended in an extraction buffer consisting of 5 mM Na_2_HPO_4_, 1 mM monothioglycerol, and 10% v v^−1^ glycerol (pH 7.4). The suspension was centrifuged for 45 min at 100 000 × **g** to obtain a supernatant, that is homogenate, which was divided into aliquots and stored at −70°C until use.

### Determination of cystatin C

Cystatin C concentrations were determined by enzyme-linked immunosorbent assay (KRKA d.d., Novo mesto, Slovenia). Recombinant human cystatin C was expressed in *Escherichia coli* as described (Cimerman *et al*, 1999) and used for the immunisation of animals and the preparation of calibration curves. Rabbit polyclonal antibody (IgG), purified from antisera by affinity chromatography on immobilised cystatin C, was used as capture antibody and murine 1A2 monoclonal antibody, conjugated to horseradish peroxidase, was used for detection (Kos *et al*, 2000a). Antibodies were specific for recombinant and native human cystatin C, and recognised free inhibitor and cathepsin-inhibitor complexes. Crossreactivity between closely related inhibitors of cysteine proteinases (human stefins A and B) was excluded by immunoblot and ELISA.

Linearity and recovery of ELISA was tested as described (Kos *et al*, 2000a). A microplate reader was used to measure the absorbance (SLT Rainbow, Salzburg, Austria) in ELISA. The concentration of cystatin C was expressed in ng mg^−1^ of total protein (ng mgp^−1^).

Tumour samples at 1 : 20 dilution and normal tissue samples at 1 : 10 dilution were added to the wells of a microtitre plate precoated with rabbit anticystatin C IgG. After 2 h of incubation at 37°C, the wells were washed and murine monoclonal 1A2 anticystatin C antibody, which was purified by the affinity chromatography on Protein A-Sepharose and conjugated subsequently with horseradish peroxidase, was added. After a further 2 h of incubation at 37°C, 3,3,4,4-tetramethyl benzidine (Sigma, St Louis, MO, USA) in the presence of hydrogen peroxide was added. The amount of degraded substrate, as a measure of bound immunocomplexed cystatin C, was visualised by absorbance at 450 nm. The cystatin C concentration was calculated from the calibration curve.

### Determination of proteins

Protein concentrations were determined according to the Bradford method (Bradford, 1976). Bovine serum albumin was used as a standard.

### Statistical analysis

The results were analysed using a PC and the SPSS statistical package (Release 10.0, SPSS Inc., Chicago, IL, USA). The difference between the median concentrations of cystatin C in matched pairs of tumour and normal tissue samples was determined by the Wilcoxon signed-rank test. The Mann–Whitney *U* test was used to test the relationship between the median values of the tumour tissue cystatin C concentrations in different groups of patients. The difference in distribution of pTNM-stages between the patients with laryngeal tumours and those with nonlaryngeal tumours was tested with a *χ*^2^ test.

Univariate analysis of the patients’ survival was carried out using the Kaplan–Meier product-limit method (Kaplan and Meier, 1958) and log-rank comparison to evaluate the difference between the survival curves (Peto *et al*, 1977). The primary end points of survival analysis were disease-free survival (DFS) and disease-specific survival (DSS). In the former case, the local and/or regional recurrence and/or systemic dissemination was considered as an event, whereas the latter was calculated by censoring deaths from disease-unrelated causes. The survival times were calculated from the date of surgery. Cystatin C concentrations were dichotomised into low and high groups after optimisation of the cutoff level, using the Critlevel method as described by Abel *et al* (1984). Multivariate analysis was performed according to Cox's proportional hazard model (Cox, 1972). All of the tests were two-sided and the results were considered significant at a probability level below 5%.

The study protocol was approved by the Medical Ethics Committee at the Ministry of Health of the Republic of Slovenia. All of the included patients gave their informed consent to voluntary participation in the study.

## RESULTS

### Distribution of cystatin C concentrations and relation to clinical and histopathological parameters

The concentrations of cystatin C in 82 tissue homogenates of tumour and corresponding nontumorous mucosa are summarised in Table 2

**Table 2:** Concentrations of cystatin C in tissue homogenates of tumours and adjacent normal mucosa

	**Cystatin C^a^**				
	**Mucosa**		**Tumour**		
**Patients**	**Median**	**Range**	**Median**	**Range**	***P*-value^b^**
*All (n=82)*	16.7	2.9–81.2	14.2	7.5–49.1	0.041
Group 1^c^ (*n*=37)	33.7^d^	8.7–81.2	13.8^e^	7.6–27.9	<0.0001
Group 2^c^ (*n*=45)	9.1^d^	2.9–26.8	18.5^e^	7.5–49.1	<0.0001
					
*With laryngeal tumours (n=40)*	24.0	5.4–79.9	16.9	7.5–49.1	0.029
Group 1^c^ (*n*=22)	37.7	11.0–79.9	12.9	7.6–27.9	0.0001
Group 2^c^ (*n*=18)	9.7	5.4–26.8	26.1	7.5–49.1	0.001
					
*With nonlaryngeal tumours (n=42)*	11.5	2.9–81.2	14.8	8.0–40.1	NS
Group 1^c^ (*n*=15)	23.8	8.7–81.2	14.5	8.0–21.9	0.0001
Group 2^c^ (*n*=27)	8.5	2.9–25.1	14.9	9.1–40.1	<0.0001

a Concentration, in ng mg^−1^ total protein.

b Mucosa *vs* tumour.

c Patients with *decreased* (Group 1) and *increased* (Group 2) concentration of cystatin C in tumour compared to mucosa.

d Mucosa: Group 1 *vs* Group 2, *P*<0.0001.

e Tumour: Group 1 *vs* Group 2, *P*=0.009.

NS=not significant.

. The cystatin C concentration was significantly (P<0.0001) decreased by a factor of 2.44 in 37 (45%) tumours and was elevated 2.04-fold in 45 cases (55%), still resulting in a 1.18-fold significant (*P*=0.041) decrease in inhibitor concentration in the total population of tumour samples compared to their control counterparts. Furthermore, the cystatin C levels in control samples in the group where an increase was observed was significantly lower than in the control group with decreased inhibitor (*P*<0.0001).

In normal mucosa samples, the cystatin C level was influenced by the site of sampling, being lower in nonlaryngeal tissue samples (i.e., from the oral cavity, oropharynx or hypopharynx) than in the laryngeal ones (11.5 *vs* 24.0 ng mgp^−1^ *P*=0.004). With regard to the cystatin C levels in tumour tissue or the distribution of downregulated and upregulated cases, no difference was found between these two major groups of tumours.

The data were further analysed with respect to the established clinical and histopathological parameters. No correlation was found between tumour cystatin C level on the one hand and, on the other hand, patients’ age and sex, histopathological tumour grade, pT-stage and the overall UICC pTNM-stage of the disease, and the presence of tumour emboli in the lymphatic vessels as determined in a histological examination of a resected tissue specimen from the neck. The tumour cystatin C level correlated inversely with pN-stage (pN_0_ *vs* pN_+_: 18.4 *vs* 14.2 ng mgp^−1^, *P*=0.047), whereas a trend of lower cystatin C levels was observed in the group with extranodal tumour extention compared to that with no extranodal spread (14.0 *vs* 17.4 ng mgp^−1^, *P*=0.069).

The analysis was repeated separately on laryngeal and nonlaryngeal tissue samples. In matched pairs of normal mucosa and tumour tissue, statistically significant (*P*=0.029) decrease in tumour cystatin C concentration was found in the group of laryngeal tumours: compared to the normal tissue counterparts, the tumour cystatin C concentration decreased by a factor of 1.42. The inhibitor decreased significantly (*P*=0.0001), 2.92-fold in 55% of tumours, and was elevated 2.69-fold in 45% of cases (Table 2). No correlation with established clinical or histopathological prognostic factors was observed in this group of tumours. Among nonlaryngeal tumour samples, no difference was found in matched pairs of normal mucosa and its tumour counterparts, and marginally significant trend of higher cystatin C concentrations was observed in early disease compared to advanced tumours (UICC pTNM stages I–II *vs* III–IV: 18.1 *vs* 13.8 ng mgp^−1^, *P*=0.052).

### Survival analysis

As of 31 December 2002 (close-out date), disease relapsed in 22 patients and 50 patients died: 20 due to disease recurrence and/or dissemination and 30 due to causes other than the treated malignant disease. In total, 32 patients were alive with no signs of the disease. The median follow-up period of all eligible patients was 4.3 years (range: 0.1–10.0 years), and was 6.5 years (5.2–10.0 years) for those alive at the last follow-up examination.

Actuarial DFS rates at 5 and 10 years were 70%, and the DSS rates were 73%. On univariate analysis, the patients with tumours involving neck nodes (pN_+_) and more advanced disease (overall UICC pTNM-stage), and those in whom extranodal tumour spread or tumour emboli were determined in the lymphatic vessels, exhibited a significantly higher risk of relapse or disease-specific death (Table 3

**Table 3:** Univariate analysis of survival

		**Disease-free survival**			**Disease-specific survival**		
**Variable**	**No. of patients**	**% at 5 years**	**95% CI**	***P*-value**	**% at 5 years**	**95% CI**	***P*-value**
*pN-stage*							
pN_0_	37	89	76–100	0.009	90	78–100	0.008
pN_+_	45	61	43–79		59	40–78	
							
*UICC pTNM-stage*							
Stage_I–III_	34	87	73–100	0.015	93	82–100	0.001
Stage_IV_	48	57	40–74		58	41–75	
							
*Extranodal spread*							
Negative^a^	52	82	69–95	0.001	89	78–100	<0.0001
Positive	30	48	28–68		45	24–66	
							
Tumour emboli							
Negative^a^	74	71	59–83	0.027	75	63–87	0.021
Positive	8	27	5–49		25	2–48	
							
*Cystatin C*							
Low	56	61	45–77	0.013	64	48–80	0.013
High	26	90	76–100		91	78–100	

a Patients with no evidence of regional metastases (i.e. pN_0_) are also included.

**Figure 1:**
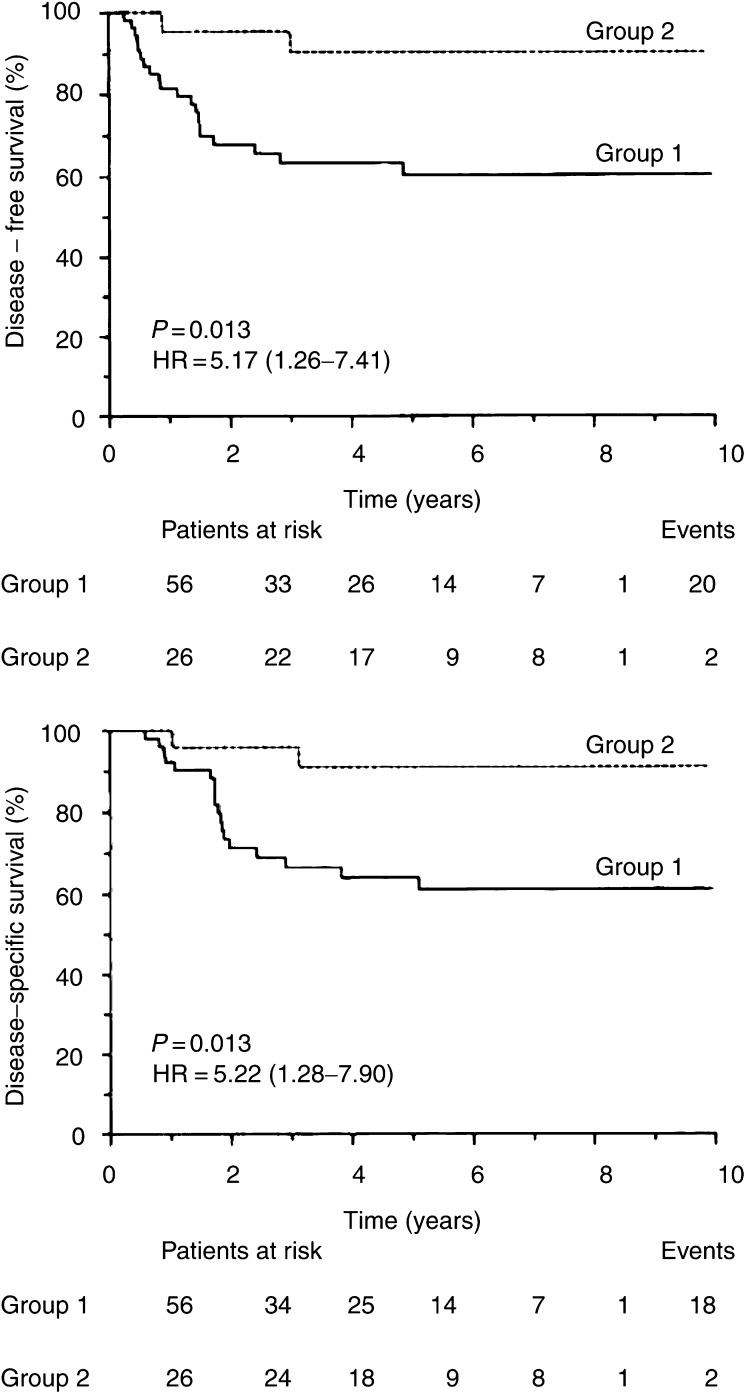
Actuarial DFS and DSS as a function of cystatin C concentration. The cutoff value was determined as described in the text. Numbers in parentheses indicate number of recurrences (or deaths)/total in each group.

CI=confidence interval.

). When using a median cystatin C concentration to classify tumours as cystatin C-low and cystatin C-high, no difference in survival was observed between the groups. However, after optimisation, the cystatin C cutoff value was found to be the 68th percentile in the group and was identical for DFS and DSS, giving in both cases a *P*-value of 0.013. As compared to the group with low tumour levels of cystatin C, patients with higher tumour contents of the inhibitor experienced significantly longer survival (Table 3, Figure 1

).

In multivariate analysis, the prognostic value of cystatin C was compared to the values of pN-stage, UICC pTNM-stage, extranodal tumour spread and the presence of tumour emboli in the lymphatic vessels. Variables were eliminated singly in a backward manner with exclusion if *P*>0.050. For DFS, pN-stage (*P*=0.040, HR=2.78, 95% CI 1.25–6.38) and cystatin C (*P*=0.043, HR=0.22, 95% CI 0.08–0.66) were retained in the final model, and for DSS, pN-stage only (*P*=0.011, HR=4.36, 95% CI 1.6–9.55). In the latter case, cystatin C turned out to be of marginal significance (*P*=0.067, HR=0.25, 95% CI 0.07–0.90). As in the same cohort of patients stefin A has been identified previously as the most powerful prognosticator for both DFS and DSS, the standardised concentrations of the latter, calculated as described in our previous report, was also introduced in Cox model (Strojan *et al*, 2000). Using the Critlevel method, the concentrations of stefin A were dichotomised into low and high groups at cutoff level, which turned to be the 25th percentile in the group. Comparing both inhibitors, stefin A proved to be stronger and the only significant predictor for survival (DFS: *P*=0.003, HR=0.27, 95% CI 0.11–0.63; DSS: *P*=0.004, HR=0.26, 95% CI 0.11–0.65).

**Figure 2:**
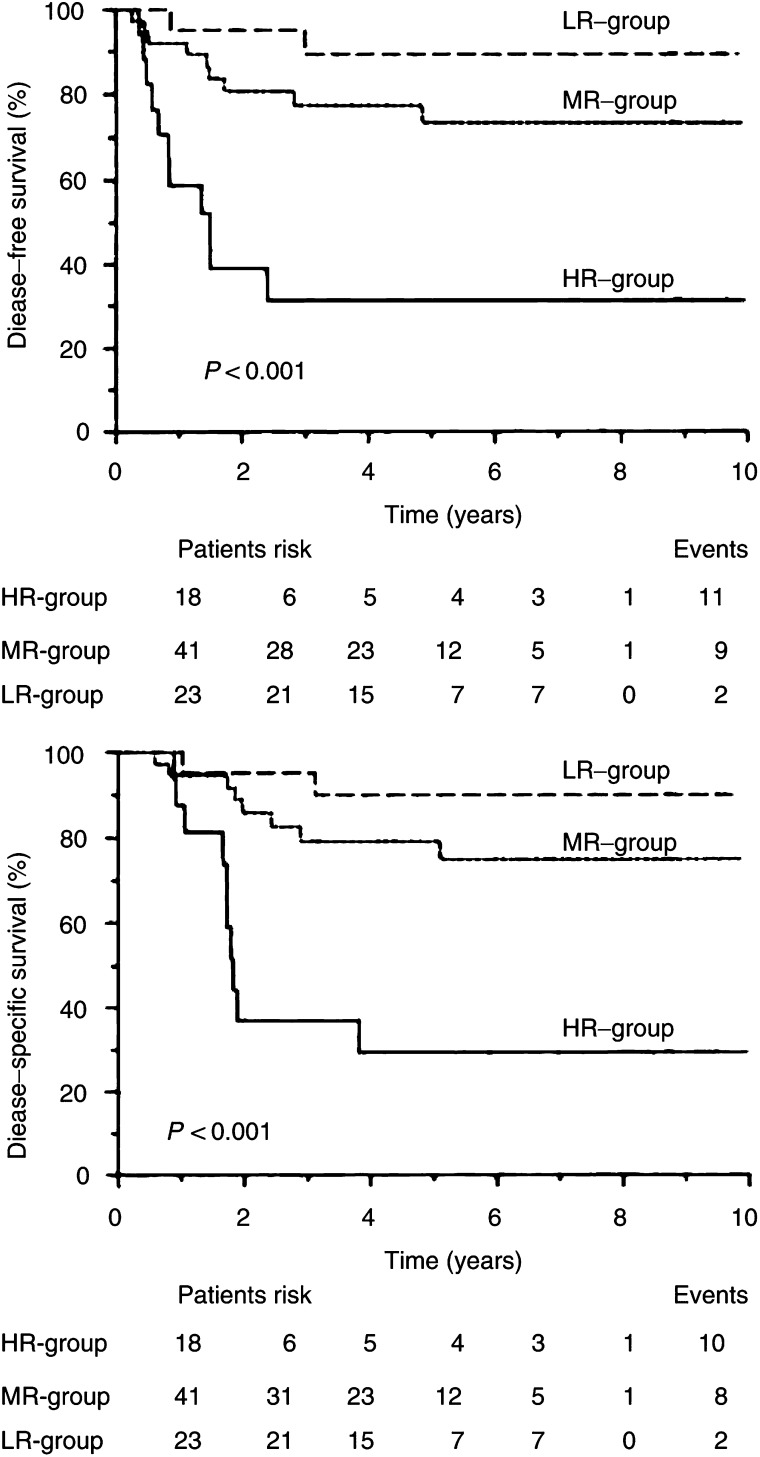
Prognostic significance of the combination of cystatin C and stefin A concentrations: DFS and DSS. The cutoff value was determined as described in the text. Numbers in parentheses indicate number of recurrences (or deaths)/total in each group (LR=low-risk group; MD=medium-risk group; HR=high-risk group).

In addition, to determine whether the combination of the two variables would increase the prognostic stratification of the patients, they were grouped as follows: high cystatin C and high stefin A – low-risk group (23 patients); high cystatin C and low stefin A, or low cystatin C and high stefin A – medium-risk group (41 patients); and low cystatin C and low stefin A – high-risk group (18 patients). We found statistically highly significant difference between the three prognostic groups for both DFS and DSS (*P*<0.0001, Figure 2

).

## DISCUSSION

An alteration of the balance between cysteine cathepsins and their endogenous inhibitors has been demonstrated to correlate with neoplastic transformation, tumour invasion and metastasis (Sloane *et al*, 1994). The tissue and serum levels of these enzymes and inhibitors in a variety of human tumours have been shown to predict the disease-free and overall survival period, and may therefore serve as prognostic factors for cancer patients. However, the investigations mainly focused on cathepsins B and L, stefins, and the most frequent cancer types, that is, carcinoma of the breast, lung and colorectum (Kos and Lah, 1998). Much less is known about cystatin C, even though it has been used in clinical research studies for more than 20 years, preferentially as a candidate marker for detection of impaired glomerular filtration rate (Newman, 2002). Based on high extracellular concentrations and inhibitory kinetics of cystatin C, it has also been shown as the most powerful extracellular inhibitor for several cysteine proteinases. Therefore, the majority of clinical work has been related to cystatin C activity and protein level determination in fluids surrounding tumours, mainly in the blood and urine (Kos *et al*, 2000b).

In the present study, a significantly lower concentration of cystatin C was found in the total population of tumour homogenates compared to normal mucosa, although two groups of patients with downregulated and upregulated cystatin C concentration were distinguished. At this point, the crucial question is whether the observed relations in cystatin C levels influenced the total intracellular proteolytic potential in the two groups. It has been shown that alterations in total inhibitory activity of cysteine proteinase inhibitors or in protein level in case of stefin A were present in malignant tissue and normal parenchyma of the breast (Lah *et al*, 1992, 1997) and lung (Knoch *et al*, 1994). In the study by Lah *et al* (1992), lowered cysteine proteinase inhibitor activity in breast carcinoma tissue (i.e., in the low-activity group) was associated with significantly higher increases of cathepsin B and cathepsin L activities than those measured in the high-activity group, indicating higher proteolytic potential in the former group. In the low-activity group, more patients developed poorly differentiated and hormone receptor-negative tumours. After a 2-year follow-up, however, recurrences were reported in the same percentage in both groups of patients. Neither in lung cancer (Knoch *et al*, 1994) nor in the present study (data not shown) was a significant difference between the two groups with respect to survival probability observed. In our study it is noteworthy that cystatin C level in control samples of the group of cystatin C positive tumours was significantly lower than in the control group with decreased inhibitor. Lah *et al* (1997), who reported on similar change for the inhibitory activity of cystatins and stefin A protein level in normal breast tissue, hypothesised that the relative increase in cystatin activity/levels was due to both an increase of cystatins in tumours and their decrease in control tissues. The latter suggested a downregulation of cystatin C in stromal tissue adjacent to the tumour, possibly induced by tumour cells.

Specifically for cystatin C, alterations were described at various levels of protein synthesis. Yano *et al* (2001) reported on the tendency of lower cystatin C mRNA expression in breast cancer tissue than in noncancerous tissue, and significantly lower expression of cystatin C relative to cathepsin B in cancerous tissue. Downregulation of cystatin C gene was found in large granular lymphocyte leukaemia (Kothapalli *et al*, 2003). On the contrary, in squamous-cell lung cancer, no alteration in cystatin C protein level was described in matched pairs of normal and tumour tissue homogenates (Ebert *et al*, 1997; Køepela *et al*, 1998) and, in colorectal cancer, a similar cystatin C mRNA level was found in cancerous and noncancerous tissue, which tended to show an inverse correlation with the cathepsin B levels (Hirai *et al*, 1999). The possibility of a similar number of pairs with equally increased and decreased cystatin C mRNA or protein level in these studies, resulting in the same average, could not be excluded.

The correlation between cysteine proteinases, their inhibitors and tumour malignancy is likely to be a qualitative rather than a quantitative one. Thus, the proteolytic activity in tumours might be affected not only by the alterations in synthesis but also in processing, subcellular localisation and secretion of the enzymes and their inhibitors. As a result, in breast cancer, the cathepsin B and L activities were found to be much more increased than the respective protein levels, suggesting that the increase in activity was not entirely due to the induction of the respective proteins (Lah *et al*, 1997). The changes in molecular structure of inhibitors may decrease the binding affinity for the target enzymes, as described by Lah *et al* (1989) for stefin A in human sarcoma. In lung tumour tissue, cathepsin B was found to be more resistant to inactivation by E-64 than cathepsin B from control lung tissue (Křepela *et al*, 1995). Furthermore, it has been shown that the serum level of cathepsin B/cystatin C complexes was significantly decreased in patients with malignant lung tumours than in healthy controls (Zore *et al*, 2001). Additionally, higher total concentrations of cystatin C found in sera of patients with lung, colorectal and melanoma cancer (Kos and Lah, 1998) suggested enhanced secretion of cystatin C from tumour cells, increasing at the same time the intracellular proteolytic potential of cysteine proteases. Association of enhanced secretion of cystatin C with invasive potential has been found in various cell lines (Kos *et al*, manuscript in preparation).

A large body of literature has been accumulated to suggest that cystatin C participated not only in the transformation of cells to a malignant state but also in tumour growth, invasion and metastasis. In cystatin C transfected B16 melanoma cells, the overexpression of inhibitor resulted in the inhibition of melanoma cell mobility and of the ability to penetrate artificial matrices by about 50% (Sexton and Cox, 1997), as well as in the suppression of metastasis by at least 90%, compared to controls (Cox *et al*, 1999). Similarly, the overproduction of active recombinant cystatin C resulted in a pronounced reduction in Matrigel invasion of murine squamous carcinoma cells (Coulibaly *et al*, 1999). In human glioblastoma cells, inverse correlation between cystatin C and tumour grade was observed (Konduri *et al*, 2002). The sence-cystatin C transfected cells were also markedly less invasive than the control cells and, in nude mice, did not form tumours upon intracerebral injection. Furthermore, it was suggested that cystatin C and cathepsin B interaction may participate in the modulation of the invasive phenotype of human colonic tumours (Corticchiato *et al*, 1992). In the mouse lymphosarcoma model, the cystatin C concentrations in the plasma and other tissues reliably reflected the changes in tumour volume following cytotoxic therapy (Poteryaeva *et al*, 2001). In our study, we observed inverse correlation between the tumour cystatin C level and more aggressive forms of the disease (i.e., involved neck nodes, extranodal tumour spread), which also links the alterations in cystatin C expression with the invasive behavior of SCCHN. The fact that the intracellular inhibitors of cysteine proteases regulate the degradation of extracellular matrix, a crucial step in invasion process, is apparently contradictive. However, it has been shown that the extracellular matrix can be internalised and partially degraded in the tumour cells (Szpaderska and Frankfater, 2001). The role of intracellular fraction of cystatin C in regulating this process has to be distinct from those of stefins A and B due to different subcellular localisation and inhibitory profile against cysteine proteases.

There is good evidence that all nucleated cells constitutively express cystatin C. However, variability in the degree of cystatin C gene expression in different tissues, which could be further influenced by several factors, provides evidence for the differential regulation of cystatin C expression in different tissues (Newman, 2002). In the present study, the results of cystatin C measurements in normal mucosa were influenced by the site of sampling. Cystatin C levels were significantly lower in the homogenates prepared from nonlaryngeal tissue samples than in the homogenates from the laryngeal tissue. Thus, considering the inherent variations in the regulation of proteolytic pathways between individual subsites inside the upper aerodigestive tract, at least two groups of tissues should be distinguished in further studies.

Concerning the results on survival in our study, two points should be stressed. To our knowledge, this is the only study evaluating the prognostic significance of cystatin C in SCCHN and no data are available at the moment for comparison. Second, the study population was quite homogenous in respect to the selection of the patients (all had operable tumours) and therapy. In multivariate analysis, only the prognostic power of the pN-stage of disease outweighs that of cystatin C. However, when comparing the prognostic strength of cystatin C with that of stefin A, which emerged as the most significant prognostic factor in our previous study analysing the same population of patient, cystatin C lost its significant prognostic power for both DFS and DSS. In addition, the combination of the two inhibitors, cystatin C and stefin A, could further stratify the risk of adverse event as was the case with stefin B and cathepsin B in colorectal cancer (Kos *et al*, 2000a).

The correlation between high levels of tumour cystatin C and the longer survival of our patients concurs with the concept of protective role of high levels of cysteine proteinase inhibitors in tissue homogenates. This concept has been proposed following the survival results in the carcinoma of the breast (Lah *et al*, 1997), lung (Knoch *et al*, 1994; Ebert *et al*, 1997), and head and neck (Strojan *et al*, 2000). Two studies apparently contradict this assumption, specifically regarding cystatin C. In colorectal cancer (Kos *et al*, 2000a) and lung cancer (Kos *et al*, unpublished results), the patients with high serum levels of cystatin C exhibited a significantly higher risk of death than those with lower levels of inhibitor, whereas a decreased metastatic spread was found in cystatin C deficient mice compared to wild-type mice (Huh *et al*, 1999). As mentioned above, alterations in secretion may result in higher extracellular and lower intracellular levels of cystatin C and, therefore, the reverse correlation of serum cystatin C with patients’ survival is to be expected. On the other hand, one has to be aware that cysteine proteases and consequently their inhibitors are also involved in biological processes other than tissue remodelling during the progression of primary tumours, such as the regulation of inflammatory and immune responses (Chapman *et al*, 1997) or apoptosis (Jaattela, 1999), so that the lack of cystatin C at the systemic level may lead to a lower metastatic spread compared to wild-type animals.

In conclusion, our data indicate that cystatin C is implicated in the invasive behavior of SCCHN. The variations in regulation of proteolytic pathways seem to be the inherent characteristic of individual subsites inside the upper aerodigestive tract, which should be considered in further studies. Moreover, the protective role of high levels of cystatin C in tissue homogenates was suggested, as it had been proposed for some other cysteine proteinase inhibitors by the survival results in breast and lung cancer as well as in SCCHN. When comparing the prognostic strength of cystatin C to that of stefin A, the latter emerged as significantly more reliable predictor for survival.
